# Inhibition of Elongation Factor-2 Kinase Augments the Antitumor Activity of Temozolomide against Glioma

**DOI:** 10.1371/journal.pone.0081345

**Published:** 2013-11-26

**Authors:** Xiao-yuan Liu, Li Zhang, JianPing Wu, Lei Zhou, Yi-Jie Ren, Wei-Qiong Yang, Zi-Jun Ming, Bo Chen, Jianrong Wang, Yi Zhang, Jin-Ming Yang

**Affiliations:** 1 Department of Pharmacology, College of Pharmaceutical Sciences, Cyrus Tang Hematology Center, Affiliated Changshu Hospital, Soochow University, Suzhou, Jiangsu, China; 2 Department of Pharmacology and The Penn State Hershey Cancer Institute, The Pennsylvania State University College of Medicine, Hershey, Pennsylvania, United States of America; University of Chicago, United States of America

## Abstract

**Background:**

Glioblastoma multiforme (GBM), the most common form of brain cancer with an average survival of less than 12 months, is a highly aggressive and fatal disease characterized by survival of glioma cells following initial treatment, invasion through the brain parenchyma and destruction of normal brain tissues, and ultimately resistance to current treatments. Temozolomide (TMZ) is commonly used chemotherapy for treatment of primary and recurrent high-grade gliomas. Nevertheless, the therapeutic outcome of TMZ is often unsatisfactory. In this study, we sought to determine whether eEF-2 kinase affected the sensitivity of glioma cells to treatment with TMZ.

**Methodology/Principal Findings:**

Using RNA interference approach, a small molecule inhibitor of eEF-2 kinase, and *in* *vitro* and *in* *vivo* glioma models, we observed that inhibition of eEF-2 kinase could enhance sensitivity of glioma cells to TMZ, and that this sensitizing effect was associated with blockade of autophagy and augmentation of apoptosis caused by TMZ.

**Conclusions/Significance:**

These findings demonstrated that targeting eEF-2 kinase can enhance the anti-glioma activity of TMZ, and inhibitors of this kinase may be exploited as chemo-sensitizers for TMZ in treatment of malignant glioma.

## Introduction

Glioblastoma multiforme (GBM) is a common and highly aggressive form of malignant brain tumor. The lethality of this malignancy is mainly due to the high invasiveness and high proliferation of glioma cells. The current strategy for the treatment of GBM is general palliative treatment, including standard chemotherapy, surgical palliative resection and focal radiotherapy [1]. Nevertheless, GBM often exhibits a high resistance to chemotherapy and radiotherapy. For instance, temozolomide (TMZ), an alkylating agent often used in conjunction with radiotherapy in treatment of GBM [2], displays limited efficacy in many cases. A recent study reported that 60-75% of patients with glioblastoma derived no benefit from treatment with TMZ [3,4]. For patients with recurrent anaplastic gliomas, more than 50% of patients failed with TMZ treatment [3]. It has been known that cellular resistance to TMZ involves alterations of DNA repair pathways and factors, including the DNA repair protein O^6^-methylguanine-DNA methyltransferase (MGMT) [5], DNA mismatch repair (MMR) system [6], and the alkylpurine-DNA-N-glycosylase (APNG; also known as DNA methylpurine-N-glycosylase [MPG]) [7]. In addition, several kinases such as protein kinase C (PKC), protein kinase A (PKA) and calcium/calmodulin-dependent protein kinase II (CaMK II) are also known to contribute to malignant phenotypes of GBM [8–10]. We have been investigating the roles and implications of eukaryotic elongation factor-2 kinase (eEF-2 kinase, also known as Ca^2+^/calmodulin-dependent protein kinase III), a critical enzyme that controls protein translation and is up-regulated in glioma and several other types of human cancer [11–13]. We and others reported that through various pathways and mechanisms, the expression and activity of eEF-2 kinase favors glioma cell survival and invasion [11,14,15] and modulates sensitivity of tumor cells to therapeutic agents such as deoxyglucose [16], velcade and curcumin [17], MK-2206 [18], and Trail [19]. In this study, we determined the effects of targeting eEF-2 kinase on the anti-glioma efficacy of TMZ, and found that combined treatment of TMZ with an inhibitor of eEF-2 kinase could achieve better therapeutic outcome.

## Materials and Methods

### Reagents and antibodies

Temozolomide and dimethyl sulfoxide (DMSO) were purchased from Sigma (St Louis, MO); 1-Hexadecyl- 2-methyl-3-(phenylmethyl)-1H-imi-dazolium iodide (NH125) was obtained from Tocris Bioscience (St. Louis, MO); the antibodies to phospho-eEF2, eEF-2, casepase-3, PARP, and LC3B, were purchased from Cell Signaling Technology (Danvers, MA); rabbit polyclonal anti-eEF2 kinase antibody was obtained from Novus Biologicals (Littleton, CO); p62 was purchased from Enzo Life Sciences (Plymouth

Meeting, PA); β-actin antibody was obtained from Santa Cruz Biotechnology Inc (Santa Cruz, CA); eEF-2 kinase-siRNA and control siRNA were synthesized by Shanghai Gene-Pharma Co. (Shanghai, China); the Cell Counting Kit-8 (CCK-8) was purchased from DojinDo Molecular Technologies, Inc. (Rockville, MA); the Annexin V-FITC apoptosis detection kit and Matrigel were purchased from BD Biosciences (San Diego, CA); the Pierce BCA Protein Assay Kit was obtained from Thermo Scientific Corp (Hudson, New Hampshire); oligofectamine reagent was purchased from Invitrogen Corp (Carlsbad, CA); other Western blot reagents were obtained from Bio-Rad Laboratories (Hercules, CA). All cell culture products were purchased from Invitrogen Corp.

### Cell lines and culture

The human glioma cell lines U251 and LN229 were originally purchased from the Cell Bank of Shanghai Institutes for Biological Sciences (Shanghai, China). The normal human astrocyte cell line, SVGp12, was originally purchased from The American Type Culture Collection (ATCC);  we obtained this cell line  from Dr. James Connor (Penn State College of Medicine). The glioma cells and SVGp12 cells were cultured in DMEM supplemented with 10% fetal bovine serum, 100 units/mL penicillin, and 100 µg/mL streptomycin. Cells were maintained at 37°C in a humidified atmosphere containing 5% CO_2_ and 95% air.

### siRNA transfection and drug treatment

siRNA targeting eEF-2 kinase and a control siRNA were synthesized by Shanghai Gene-Pharma Co. (Shanghai, China). For transfection, cells in the exponential phase of growth were plated in 60-mm tissue culture dishes at 5×10^5^cells per dish, grown for 24 h, and then transfected with siRNA using Oligofectamine and OPTI MEMI-reduced serum medium (Invitrogen). Transfection of siRNA was performed according to the manufacturer’s protocol.

NH125 was reconstituted in DMSO (0.5 mmol/L) as a stock solution; temozolomide was reconstituted in DMSO (100 mmol/L) as a stock solution. LN229 and U251 cells were treated with various concentrations of TMZ in the presence or absence of NH125 (0.5μM), or with or without silencing of eEF-2 kinase expression. 

### Cell viability assay

Cell viability was measured by CCK-8 assay. Brieﬂy, LN229 and U251 cells were plated in 96-well plates (5×10^3^ cells/well) and subjected to different treatments. Following a 24h incubation at 37°C in a humidiﬁed atmosphere containing 5% CO_2_/95% air, 5μl of CCK-8 reagent was added to the cells. The plates were read at 450 nm on a multiscan plate reader after a 2h incubation.

### Colony-formation assay

LN229 and U251 cells subjected to different treatments were plated in 35-mm tissue culture dishes. Following incubation at 37°C in a humidified atmosphere containing 5% CO2/95% air for 10 days, cells were stained with 1% methylene blue in 50% methanol and colonies (>50 cells) were counted.

### Wound healing *assay*

Cellular ability to migrate was measured by a wound migration assay. Briefly, LN229 and U251 cells were plated onto 6-well tissue culture plates (5×10^5^/well) and cultured in medium containing 10% FBS to nearly confluent monolayers. Cell monolayers were then carefully wounded using a 10μL sterile pipette tip, and any cellular debris was removed by washing with PBS. The wounded monolayers were then subjected to the drug treatments. At the end of treatments, the cells were photographed using a phase-contrast microscope (Nikon) and analyzed for the distance migrated by the leading edge of the wound at 0 and 24h. The experiments were performed in triplicate wells and repeated at least three times.

### Transwell Invasion assay

Cell ability to invade was measured by a transwell invasion assay in modified Boyden chambers with filter inserts with 8-μm pores in 24-well plates (Corning, Tewksbury, MA ). as described previously [15]. 

### Western blot analysis

Fifty µg of protein diluted in NuPAGE-sample buffer containing reducing reagents were denatured at 95°C for 5 min and electrophoretically separated by 10-15% sodiumdodecyl sulfate-polyacrylamide gel electrophoresis (SDS-PAGE). Proteins were transferred onto nitrocellulose membranes and the membranes were blocked in 5% BSA/TBST for an hour at room temperature. The blots were probed with the respective antibodies. Protein signals were analyzed using a computer-assisted image Odyssey Western Blot analysis system and the Image J gel analysis software.

### Annexin V -FITC staining

Annexin V staining was performed using the Annexin V-FITC apoptosis detection kit (Sigma, St Louis, MO), according to the manufacturer’s instructions. The cells positive for Annexin V-FITC and/or PI were analyzed using a BD FACS flow cytometer (San Diego, CA).

### Animal study

Six-week-old male BALB/c nude mice were used for intracerebral implantation of glioma cells. LN229 cells (1 ×10^5^ cells in 15 μL of DMEM medium) were injected into the brains at 4 mm depth under anesthesia with chloralic hydras (4%, 2mL/kg, i.p.). Three days after inoculation, the mice were randomly divided into 4 groups (15 mice per group). Treatments were begun on day 4, according to the following regimens: TMZ groups: 8 mg/kg, i.p., on days 1-10; NH125, 1 mg/kg on days 1, 3, 5, 7, 9 and 11. Control groups: 0.5% DMSO in saline, on the identical schedule. Animal body weight and physical signs were monitored daily during the experiments. The mice were housed in a temperature-controlled and light-controlled environment. On day 17, the mice were euthanized, and the brains were ﬁxed in 10% buffered formalin, embedded in parafﬁn and then stained with hematoxylin-eosin (H&E) and TUNEL staining kits. The slides were photographed using a phase-contrast microscope. All animal experiments were performed according to the protocols approved by the Institutional Animal Care and Use Committee of Soochow University.

### Statistical analysis

Student’s *t*-test was used for direct comparison; the one-way analysis of variance (ANOVA) with the post-test was used for multiple comparisons. The statistical significance limit was set at p < 0.05.

## Results

### Combined treatment with TMZ and eEF-2 kinase inhibitors more strongly reduces the growth of glioma cells

**Figure 1:**
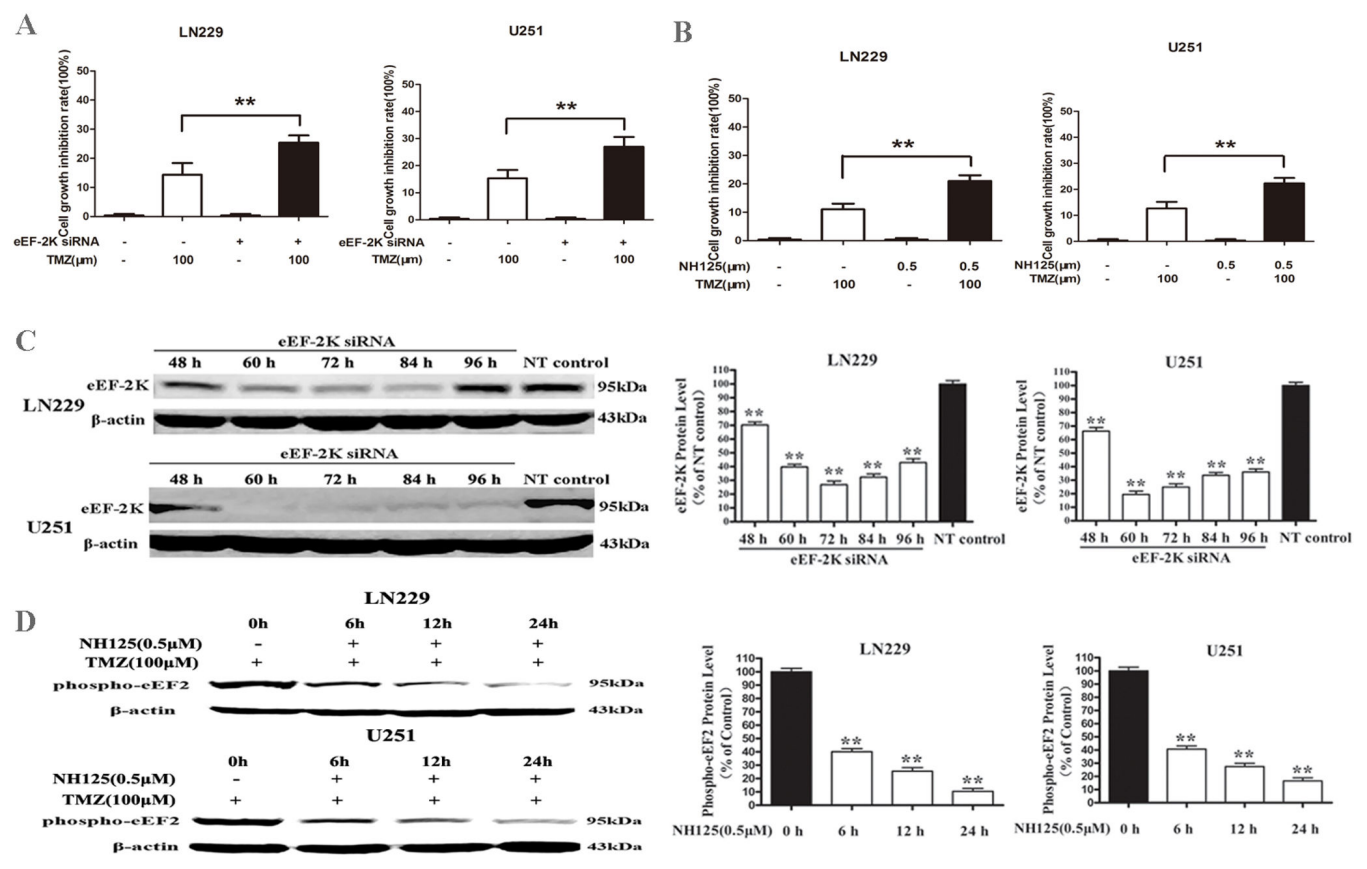
Effect of co-treatment with TMZ and eEF-2 kinase inhibitors on growth of glioma cells. (**A**) Human glioma cell lines LN229 and U251 with or without silencing of eEF-2 kinase expression were plated in 96-well plates (5×10^3^/well) and treated with TMZ (100 μM) at 37°C for 24 h; (**B**) LN229 and U251 cells were plated in 96-well plates (5×10^3^/well) and treated with TMZ (100 μM) in the presence or absence of 0.5μM of NH125 at 37°C for 24 h. Cell viability was measured by CCK-8 assay. (**C**) LN229 and U251 cells were transfected with an eEF-2 kinase-targeted siRNA or a non-targeting control siRNA (NT control). At different time points, expression of eEF-2 kinase was analyzed by Western blot,. β-actin was used as a loading control. (**D**) LN229 and U251 cells were treated with NH125 (0.5μM) or vehicle for various periods of time. At the end of treatment, the level of phospho-eEF2 (Thr56) was examined by western blot. β-actin was used as a loading control. Each bar represents mean ± SD of triplicate determinations; results shown are the representative of three identical experiments; data are expressed as the percentage of the controls. ** *p* < 0.01.

To evaluate whether inhibition of eEF-2 kinase would enhance the cytotoxicity of TMZ, we selected a sub-maximal doses of TMZ (100μM) and the eEF-2 kinase inhibitor, NH125, or eEF-2 kinase-targeted siRNA. We observed that combined treatment of TMZ with an eEF-2 kinase-targeted siRNA (Figure 1A) or the eEF-2 kinase inhibitor NH125 (0.5μM) (Figure 1B) significantly increased the growth-inhibitory effect of TMZ on the human glioma cell lines LN229 and U251, as compared with treatment with TMZ alone. The glioma cells transfected with the eEF-2 kinase-targeted siRNA showed a 70%–80% (LN229) and 85-90% (U251) reduction in eEF-2kinase protein expression at 60h after transfection, as compared with the cells transfected with a non-targeting siRNA (Figure 1C). LN229 and U251 cells treated with NH125 (0.5μM) showed a significant decrease in the activity of eEF-2 kinase, as reflected in a time-dependent reduction in the level of phospho-eEF2 protein, as compared with the cells treated with vehicle (Figure 1D). Long-term clonogenic assay showed the similar synergistic effect of eEF-2 kinase inhibitors with TMZ on inhibition of tumor cell growth and proliferation (Figure S1). The combination of TMZ with NH125 did not cause cytotoxicity in the normal human astrocytes, SVGp12 (Figure S2).

### Combined treatment with TMZ and eEF-2 kinase inhibitors more effectively inhibits the migration and invasion of glioma cells

**Figure 2:**
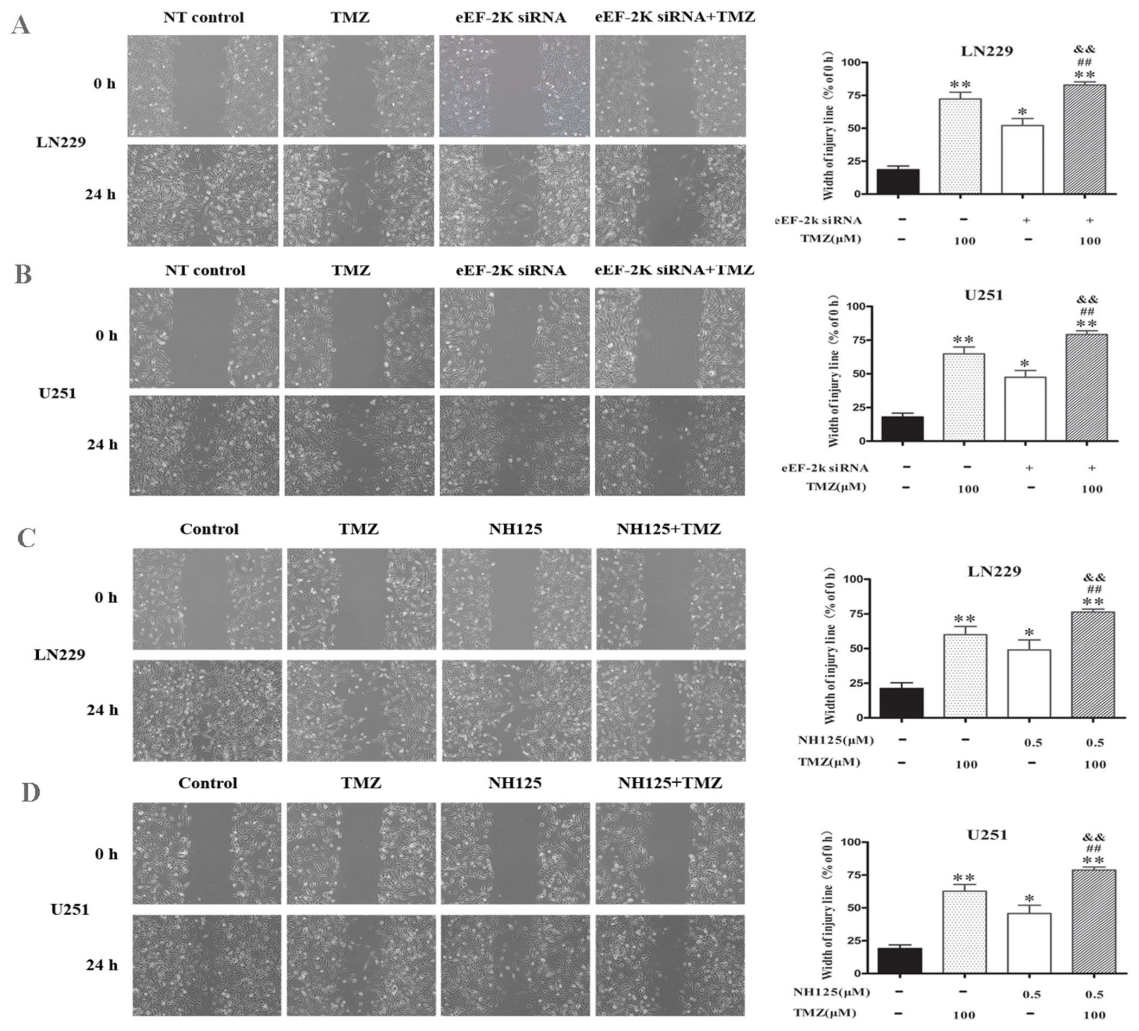
Effect of co-treatment with TMZ and eEF-2 kinase inhibitors on migration of glioma cells. (**A** and **B**) LN229 and U251 cells were transfected with an eEF-2 kinase-targeted siRNA or a non-targeting control siRNA (NT control), and then plated in 6-well tissue culture dishes (5×10^5^/well). An injury line was made on the confluent monolayer of cells and then treated with the indicated concentration of TMZ at 37°C for 24 h. (**C** and **D**) LN229 and U251 were plated in 6-well tissue culture plates (5×10^5^ /well). When cells were confluent, an injury line was made on the monolayer of cells and treated with the indicated concentration of TMZ in the presence or absence of NH125(0.5μM) at 37°C for 24h. Cell migration was observed with a light microscope and imaged at 0 and 24 h. Width of the lines was measured and the mean ± SD from three independent experiments were shown. * *p* < 0.05, ** *p* < 0.01, vs Control/ NT control; ^#^ *p* < 0.05, ^##^ *p* <0.01, vs NH125 or eEF-2 kinase siRNA; ^&^ *p* <0.05, ^&&^ *p* <0.01, vs TMZ.

**Figure 3:**
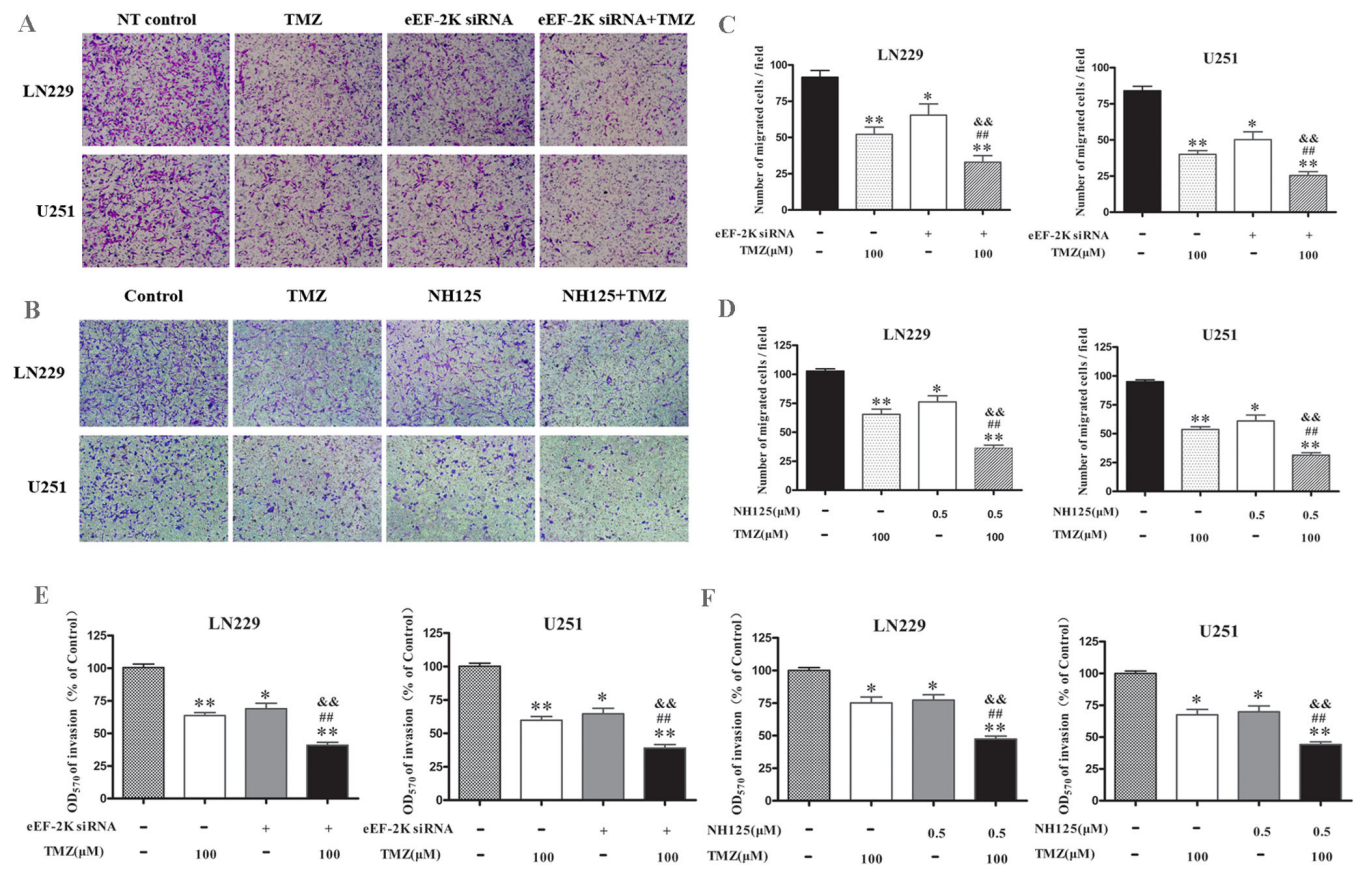
Effect of co-treatment with TMZ and eEF-2 kinase inhibitors on invasion of glioma cells. (**A**) LN229 and U251 cells transfected with an eEF-2 kinase-targeted siRNA or a non-targeting control siRNA (NT control) were plated onto 6-well plates (5×10^5^/well) and treated with the indicated concentration of TMZ; (**B**) LN229 and U251 cells were plated onto 6-well plates (5×10^5^/well) and treated with the indicated concentration of TMZ in the presence or absence of NH125 (0.5μM). At the end of treatment, same amount of cells (1×10^5^/well) were seeded on the upper chamber of transwell coated with Matrigel. Twenty-four hour later, the number of cells migrated into the lower chambers were fixed with ice-cold methanol, stained with crystal violet, and then imaged and counted and under a microscope (**C** and **D**). (**E** and **F**) The transwell chambers were fixed with 33% ice-cold acetic acid, vibrated and read in a microplate reader at an wavelength of 570 nm (E and F). *p < 0.05, **p < 0.01, vs Control/NT control; ^#^ *p* <0.05, ^##^ *p* <0.01, vs NH125 or eEF-2K siRNA; ^&^ *p* < 0.05, ^&&^ *p* <0.01, vs TMZ.

We also determined the effects of co-treatment of TMZ with eEF-2 kinase inhibitors on migration and invasion of glioma cells using wound healing assay and transwell matrigel invasion assay, respectively. As shown in Figure 2, inhibition of eEF-2 kinase by either siRNA (Figure 2A and Figure 2B) or NH125 (Figure 2C and Figure 2D) could strengthen the inhibitory effect of TMZ on wound healing in U251 and LN229 cells. Figure 3 shows that the invasive potentials of U251 and LN229 cells were also more effectively suppressed by the co-treatment of TMZ with either an eEF-2 kinase-targeted siRNA (Figure 3A, Figure 3C and Figure 3E) or the eEF-2 kinase inhibitor, NH125 (Figure 3B, Figure 3D and Figure 3F), as compared with that in the cells treated with TMZ alone. 

### Inhibiting eEF-2 kinase blocks autophagy activated by TMZ in glioma cells

**Figure 4:**
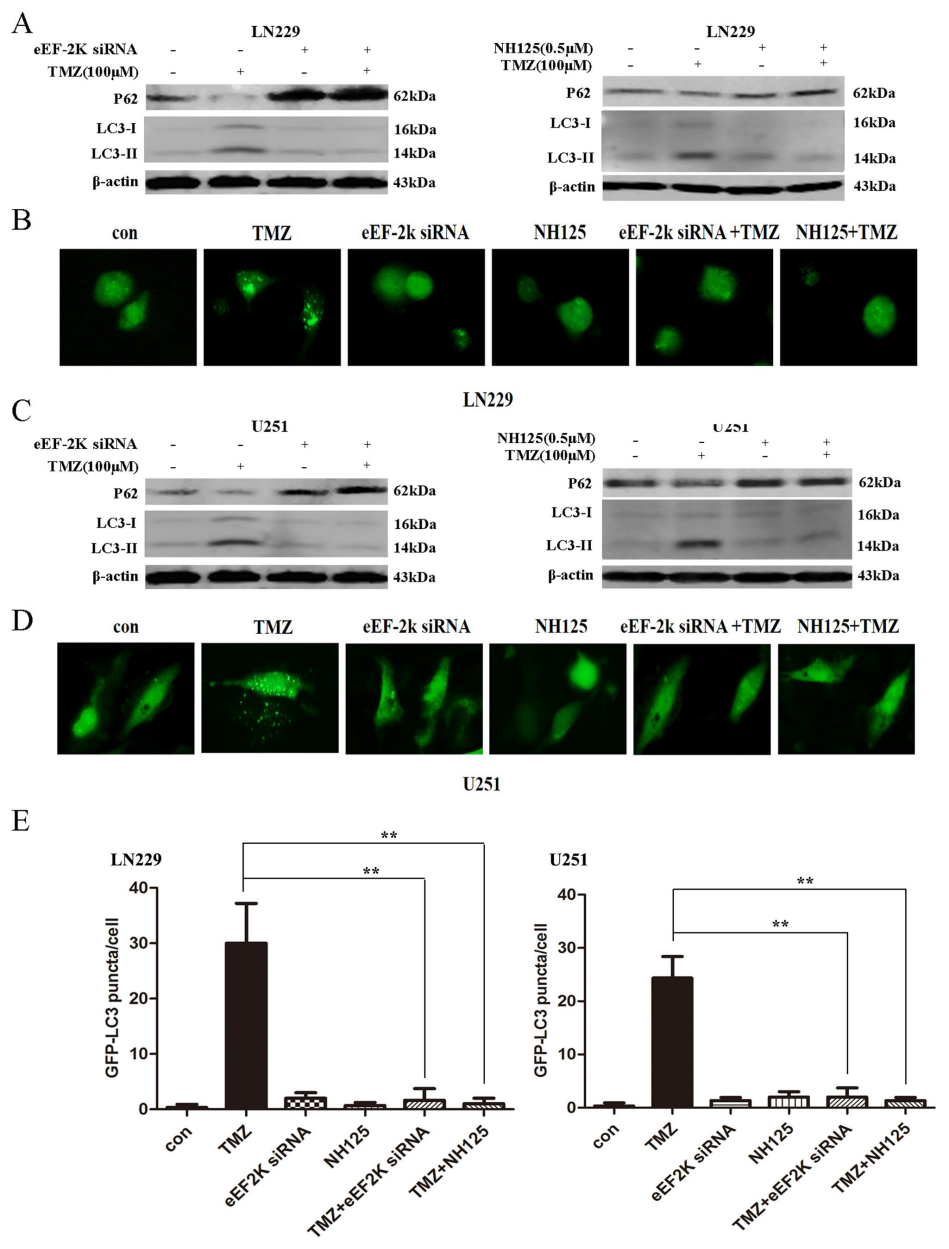
Effect of inhibiting eEF-2 kinase on the TMZ-induced autophagy activation in glioma cells. LN229 (**A** and **B**) and U251 (**C** and **D**) cells were treated with the indicated concentration of TMZ for 24h in the presence or absence of silencing of eEF-2 kinase expression or in the presence or absence of NH125. At the end of treatment, the levels of p62 and LC3-II were determined by Western blot. β-actin was used as a loading control (**A** and **C**). GFP-LC3 punctum formation was observed by microscopy. (**E**) Quantitation of the GFP-LC3 puncta was performed by counting 20 cells for each sample, and average numbers of puncta per cell were shown. **p < 0.01, *t*-test. Each bar represents mean ± SD of triplicate determinations; results shown are the representative of three identical experiments.

TMZ is known to activate autophagy in glioma cells [20], and we previously showed that inhibition of eEF-2 kinase blunted autophagy induced by various stresses [11,16,18]. Thus, we asked whether autophagy modulation is involved in the synergistic action of TMZ and eEF-2 kinase inhibitors against tumor cells. In these experiments, LN229 and U251 cells were treated with the indicated concentration of TMZ for 24h in the presence or absence of the eEF-2 kinase-targeted siRNA or NH125. At the end of treatment, autophagy was examined by Western blot analysis of the autophagy markers, LC3-II and p62, and by microscopic observation of the GFP-LC3 punctum formation. We found that inhibition of eEF-2 kinase by either siRNA or NH125 blocked the activation of TMZ-induced autophagy in both of LN229 (Figure 4A, Figure 4B and Figure 4E) and U251 (Figure 4C, Figure 4D and Figure 4E) cells, as evidenced by decreases in LC3-II level (Figure 4A and Figure 4C) and in GFP-LC3 punctum number (Figure 4B, Figure 4D and Figure 4E), and an increase in p62 level (Figure 4A and Figure 4C). These results suggest that suppression of the TMZ-induced autophagy by eEF-2 kinase inhibition may contribute to the greater cytotoxicity resulting from co-treatment of TMZ and the inhibitors of the kinase.

### Inhibition of eEF-2 kinase augments the TMZ-induced apoptosis in glioma cells

**Figure 5:**
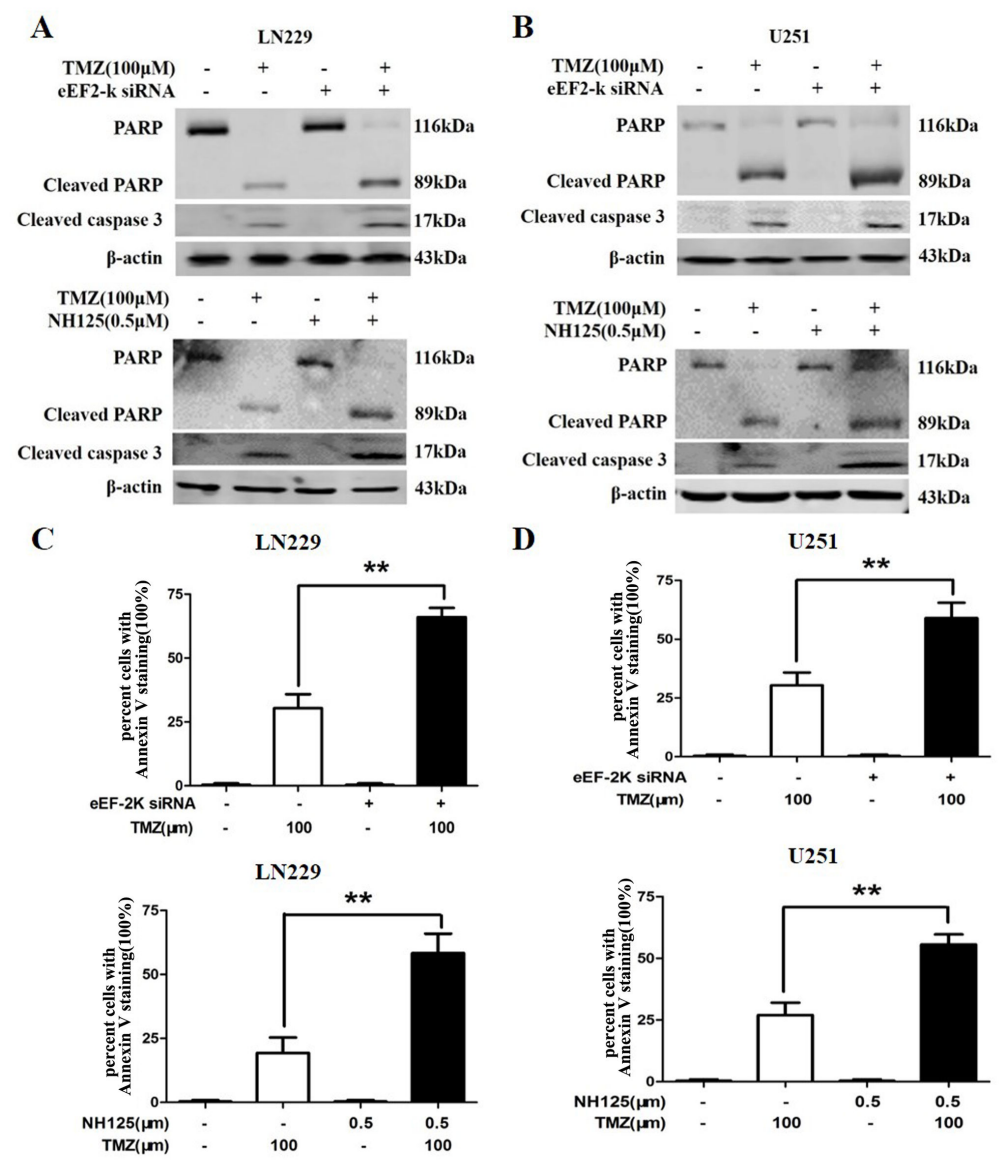
Effect of inhibiting eEF-2 kinase on the TMZ-induced apoptosis in glioma cells. LN229 and U251 cells were treated with the indicated concentration of TMZ for 24 h in the presence or absence of silencing of eEF-2 kinase expression or in the presence or absence of NH125. At the end of treatment, apoptosis was determined by Western blot of cleaved PARP and caspase-3 (**A** and **B**) and by cytometric analysis of Annexin V staining (**C** and **D**). β-actin was used as a loading control. **p < 0.01, *t*-test. Each bar represents mean ± SD of triplicate determinations; results shown are the representative of three identical experiments.

As suppression of autophagy is often accompanied by activation of apoptosis, we next measured and compared apoptosis in the glioma cells treated with TMZ alone or with both of TMZ and eEF-2 kinase inhibitors. We found that silencing of eEF-2 kinase expression or treatment with NH125 remarkably augmented the TMZ-activated apoptosis in LN229 and U251 cells, as demonstrated by the increases in the amounts of cleaved caspase-3 and PARP (Figure 5A and Figure 5B), and in Annexin V staining (Figure 5C and Figure 5D). These experiments provide further evidence for the therapeutic potential of targeting eEF-2 kinase in reinforcing the cytocidal effects of TMZ.

### Effect of the combinatorial treatment with TMZ and NH125 on glioma growth *in vivo*

**Figure 6:**
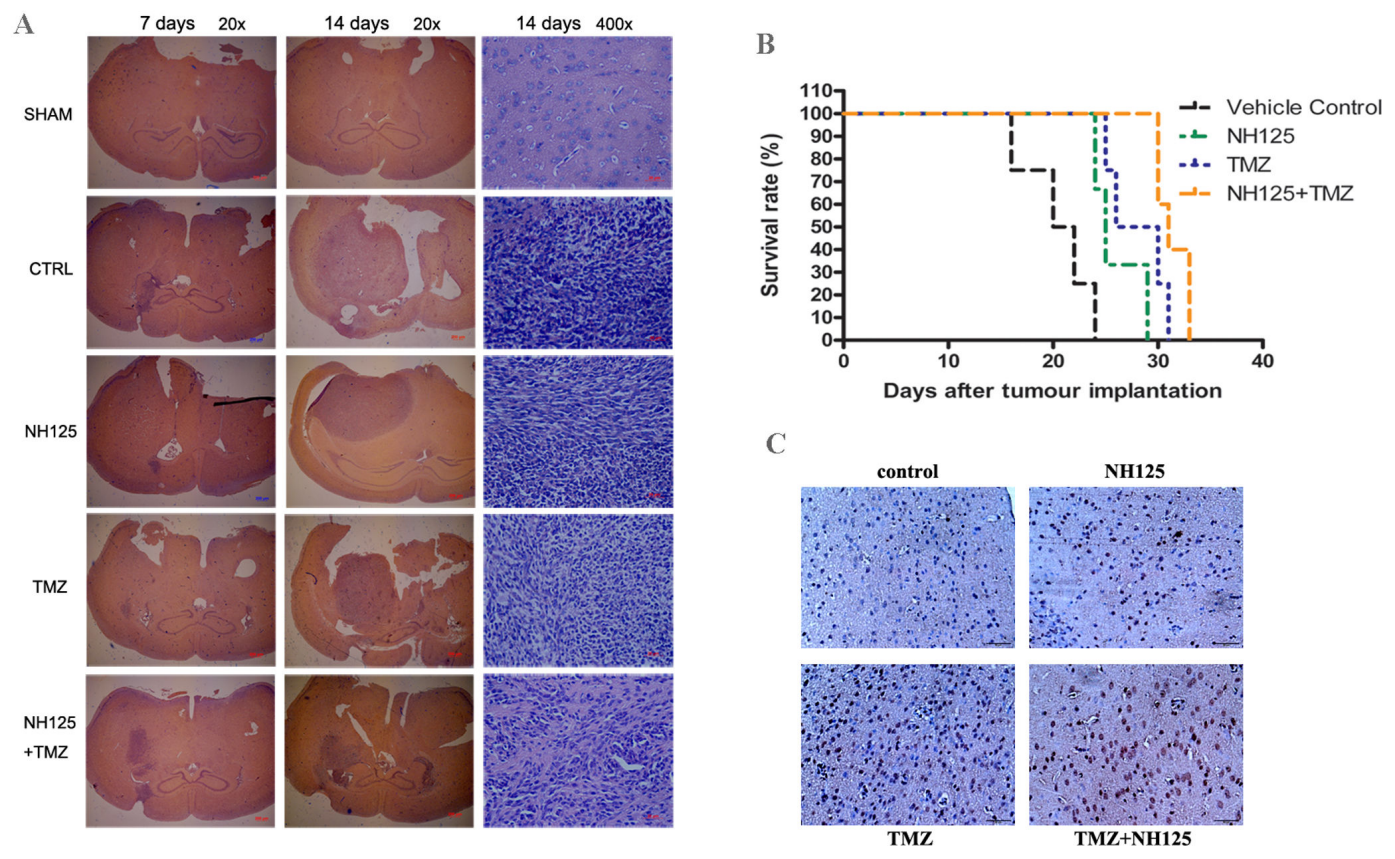
Effect of the combinatorial treatment with TMZ and NH125 on glioma growth mouse in an intracranial xenograft model. The human glioma LN229 cells were implanted into the anesthetized nude mice (5×10^6^ cells/per site) through a burr hole on the skulls. Three days later, the inoculated mice were divided into four groups (10 mice per group) and subjected to different treatments: (1) Control groups receive appropriate vehicles (0.5% DMSO in PBS); (2) TMZ (8 mg/kg) on days 1- 10, i.p.; (3) NH125 (1 mg/kg) on days 1, 3, 5, 7, 9 and 11, i.p.; (4) TMZ plus NH125. (**A**) HE staining of the paraffin sections of the tumor xenografts. (**B**) The survival curves of the tumor-bearing mice. Median survival (days): vehicle: 21days; NH125: 25 days; TMZ: 28 days; TMZ+NH125: 31 days. The pooled-variance two-tailed *t*-test showed *p* <0.05 for TMZ+NH125 vs TMZ. (**C**) TUNEL staining of the paraffin sections of the tumor xenografts.

To evaluate the *in vivo* therapeutic benefit of the combined treatment of TMZ with the eEF-2 kinase inhibitor NH125, we utilized an intracranial xenograft model of LN229 glioma cells. In these experiments, the tumor-bearing mice were either treated with vehicle, TMZ (8mg/kg) or NH125 (1mg/kg) alone, or a combination of TMZ and NH125. Figure 6A shows that TMZ and NH125 alone had tumor-inhibitory effects as compared with the vehicle. However, combination of TMZ and NH125 caused a greater inhibition of tumor growth than TMZ or NH125 alone (Figure 6A). The HE staining photograph of glioma specimens showed that this co-treatment did not fully eradicate tumors but delayed the growth of the glioma (Figure 6A). Figure 6B shows that, as compared with NH125 or TMZ mono-therapy, co-administration of TMZ with NH125 produced a better survival beneﬁt. TUNEL staining of the tumor specimens demonstrated that the numbers of cells positive for TUNEL staining were higher in the combination of TMZ and NH125 group than that in the TMZ group (Figure 6C), indicating that this combination induces more apoptotic cell death.

## Discussion

Although TMZ is commonly used in treatment of GBM, the therapeutic outcome is often unsatisfactory, due to various causes and mechanism. Strategies and approaches to reinforcing the antitumor efficacy of TMZ will make this drug more useful and beneficial to patients with malignant glioma. In this study, we explored whether targeting eEF-2 kinase, an enzyme critical in regulation of protein synthesis, could impact the effectiveness of TMZ. Our results showed that inhibiting eEF-2 kinase could strengthen the inhibitory effects of TMZ on growth, proliferation, migration and invasion of glioma cells (Figure 1, Figure 2, Figure 3 and Figure 6). We further demonstrated that the enhanced antitumor activity of TMZ resulting from inhibition of eEF-2 kinase were associated with suppression of autophagy and promotion of apoptosis (Figure 4 and Figure 5).

We previously reported that inhibition of eEF-2 kinase could render tumor cells more sensitive to AKT inhibitors [18], growth factor antagonists [21], ER stressors [17], metabolic stress [11] and glycolytic inhibitors [16], through modulating autophagy, a cellular process that can either promote cell survival or cell death. A more recent study confirms and provides further evidence for the role of eEF-2 kinase in protecting cells from nutrient deficiency and in conferring tumor cell adaptation to metabolic stress [22]. These findings suggest that eEF-2 kinase may act as an energy sensor and provides a protective mechanism against energy stress. Because TMZ is known to induce autophagy [20], we sought to determine whether inhibiting eEF-2 kinase could suppress the TMZ-induced autophagy and impact the efficacy of this chemotherapeutic drug. Similar to our previous studies, we found that inhibiting eEF-2 kinase by RNAi or chemical inhibitor can sensitize glioma cells to TMZ. We believe that this is due to the properties of eEF-2 kinase as a general regulator of protein synthesis and energy metabolism. Autophagy activation is now appreciated as a protective mechanism to cope with various types of stresses including therapeutic stress. Our results showing that inhibiting eEF-2 kinase-mediated autophagy enhanced cytotoxicity of TMZ further supports the pro-survival role of autophagy in cells stressed with therapeutic insults. In this study, we tested the effects of NH125, a small molecule inhibitor highly selective for eEF-2 kinase, as compared to other kinases such as protein kinase A, protein kinase C and calmodulin kinase II [23]. We observed in this study that NH125 shows good synergistic effects with TMZ against glioma both *in vitro* and *in vivo*. Nevertheless, we recognize that, although inhibiting eEF-2 kinase by NH125 can sensitize glioma cells to TMZ, the combination of the two agents could not cure the tumor-bearing mice. This is likely because that the regimen or dosages of the treatment was not optimal, or the amount of NH125 in the cerebral spinal fluid (CSF) was not enough. However, this “proof of concept” study provides an impetus for further investigation of eEF-2 kinase inhibitors as sensitizers of TMZ in treatment of malignant glioma. Development of better and more effective inhibitors of eEF-kinase should help facilitate this process. 

 In summary, the results of this study provide experimental evidence for the effectiveness of combined treatment with TMZ and eEF-2 kinase inhibitors in treating glioma, and suggest that this approach may be worth further exploring as a therapeutic strategy against malignant brain tumor. 

## Supporting Information

Figure S1 **Effect of co-treatment with TMZ and eEF-2 kinase inhibitors on clonogenecity of glioma cells.** LN229 (**A**) or U251 (**B**) cells were treated with 100 µM of TMZ for 24 h in the presence or absence of silencing of eEF-2 kinase expression or in the presence or absence of NH125; sixty h later, the cells were plated in 35-mm cell culture dishes and incubated for 10 days at 37°C in a humidified atmosphere containing 5% CO2/95% air. At the end of incubation, colonies were stained with 1% methylene blue in 50% methanol for 30 min, washed with water, and colonies counted. The bars are the mean ± S.D. of triplicate determinations; results shown are the representative of three identical experiments. * *p* < 0.05,** *p* < 0.01.
(TIF)

Figure S2 **Effect of TMZ, NH125 or co-treatment with TMZ and NH125 on viability of normal human astrocytes.** Normal human astrocytes, SVGp12, were treated with 100 μM of TMZ for 48 h in the presence or absence of NH125 (0.5 μM). At the end of treatment, cell viability was measured by MTT assay. Each bar represents mean ± S.D. of triplicate determinations; results shown are the representative of three identical experiments.
(TIF)
