# Supplementary figures and images for: Inhibition of Elongation Factor-2 Kinase Augments the Antitumor Activity of Temozolomide against Glioma
Source: PLoS One. 2013 Nov 26;8(11):e81345. doi: 10.1371/journal.pone.0081345 (PMC3841121; doi:10.1371/journal.pone.0081345)

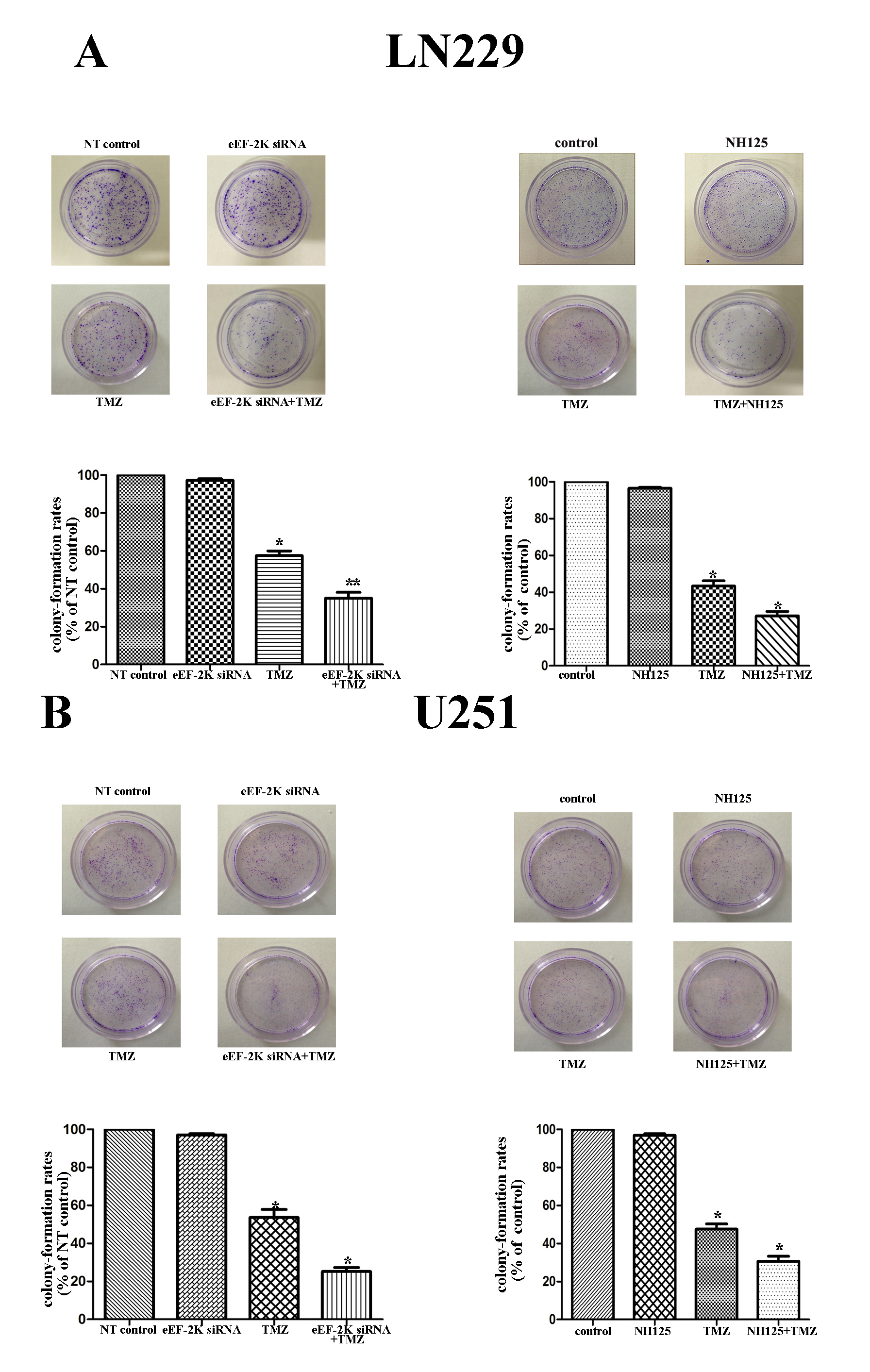

Supplement: Figure S1 — Effect of co-treatment with TMZ and eEF-2 kinase inhibitors on clonogenecity of glioma cells. LN229 (A) or U251 (B) cells were treated with 100 µM of TMZ for 24 h in the presence or absence of silencing of eEF-2 kinase expression or in the presence or absence of NH125; sixty h later, the cells were plated in 35-mm cell culture dishes and incubated for 10 days at 37°C in a humidified atmosphere containing 5% CO2/95% air. At the end of incubation, colonies were stained with 1% methylene blue in 50% methanol for 30 min, washed with water, and colonies counted. The bars are the mean ± S.D. of triplicate determinations; results shown are the representative of three identical experiments. * p < 0.05,** p < 0.01. (TIF) [file pone.0081345.s001.tif]

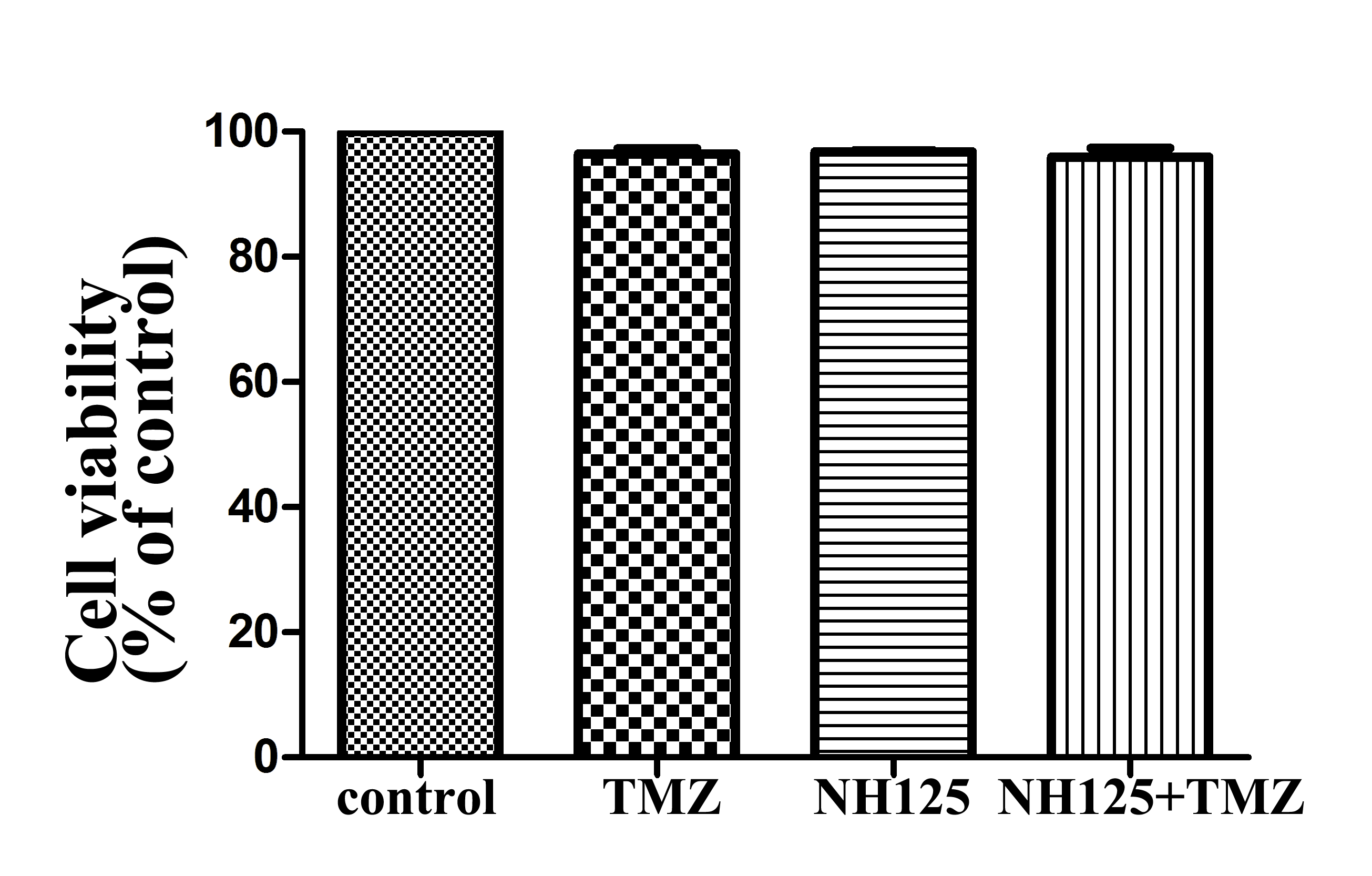

Supplement: Figure S2 — Effect of TMZ, NH125 or co-treatment with TMZ and NH125 on viability of normal human astrocytes. Normal human astrocytes, SVGp12, were treated with 100 μM of TMZ for 48 h in the presence or absence of NH125 (0.5 μM). At the end of treatment, cell viability was measured by MTT assay. Each bar represents mean ± S.D. of triplicate determinations; results shown are the representative of three identical experiments. (TIF) [file pone.0081345.s002.tif]
